# Analysis of small nucleolar RNAs in sputum for lung cancer diagnosis

**DOI:** 10.18632/oncotarget.4219

**Published:** 2015-05-20

**Authors:** Jian Su, Jeipi Liao, Lu Gao, Jun Shen, Maria A. Guarnera, Min Zhan, HongBin Fang, Sanford A. Stass, Feng Jiang

**Affiliations:** ^1^ Department of Pathology, University of Maryland School of Medicine, Baltimore, MD, USA; ^2^ Department of Epidemiology and Public Health, University of Maryland School of Medicine, Baltimore, MD, USA

**Keywords:** lung cancer, sputum, biomarkers, snoRNAs, diagnosis

## Abstract

Molecular analysis of sputum presents a noninvasive approach for diagnosis of lung cancer. We have shown that dysregulation of small nucleolar RNAs (snoRNAs) plays a vital role in lung tumorigenesis. We have also identified six snoRNAs whose changes are associated with lung cancer. Here we investigated if analysis of the snoRNAs in sputum could provide a potential tool for diagnosis of lung cancer. Using qRT-PCR, we determined expressions of the six snoRNAs in sputum of a training set of 59 lung cancer patients and 61 cancer-free smokers to develop a biomarker panel, which was validated in a testing set of 67 lung cancer patients and 69 cancer-free smokers for the diagnostic performance. The snoRNAs were robustly measurable in sputum. In the training set, a panel of two snoRNA biomarkers (snoRD66 and snoRD78) was developed, producing 74.58% sensitivity and 83.61% specificity for identifying lung cancer. The snoRNA biomarkers had a significantly higher sensitivity (74.58%) compared with sputum cytology (45.76%) (*P* < 0.05). The changes of the snoRNAs were not associated with stage and histology of lung cancer (All *P* >0.05). The performance of the biomarker panel was confirmed in the testing cohort. We report for the first time that sputum snoRNA biomarkers might be useful to improve diagnosis of lung cancer.

## INTRODUCTION

Lung cancer is the number one cancer killer in the USA and worldwide [1]. Tobacco smoking is the major cause of lung cancer [1]. Non-small lung cancer (NSCLC) accounts for 85% of all lung cancer cases. The overall 5-year survival rate for stage I NSCLC patients who are typically treated with surgery remains up to 83%. In contrast, only 5-15% and less than 2% of patients with stage III and IV NSCLC are alive after five years [1]. These statistics provide the primary rationale to improve NSCLC early detection. Furthermore, a NCI-National Lung Screening Trail (NLST) showed that the early detection of lung cancer by using low-dose computed tomography (LDCT) significantly reduced the mortality [2]. However, LDCT has limited ability to differentiate malignant from benign pulmonary nodules (PNs), presenting a major clinical challenge for lung cancer early detection [3]. Therefore, there is an urgent need for developing approaches that can improve diagnosis of NSCLC [3].

Sputum is a noninvasively and easily accessible body fluid that contains exfoliated bronchial epithelial cells [4]. Sputum cytology can identify morphological abnormalities of bronchial epitheliums of lung cancer patients [5]. Yet it has a poor sensitivity for diagnosis of lung cancer [5]. Molecular study of sputum could detect the cells containing lung tumor-associated molecular aberrations, thus providing a noninvasive approach for diagnosis of lung cancer [5]. Numerous sputum biomarkers have been identified. However, none has been acceptable for clinical utility in diagnosis of lung cancer [5].

Non-coding RNAs (ncRNAs) can regulate a wide range of biological processes, including chromatin remodeling, gene transcription, mRNA translation, and protein function [6]. ncRNAs can be divided into three categories based on length or number of nucleotides (nts) [7]. 1) Small ncRNAs are 17-30 nts in length and include microRNAs (miRNAs), piwi-interacting RNAs, and transcription initiation RNAs. 2) Middle-size ncRNAs are typically 20 and 200 nts in length and mainly consist of small nucleolar RNAs (snoRNAs). 3) Long ncRNAs (lncRNAs) are over 200 nts, which comprises several well-characterized ncRNA, such as MALAT1 and HOTAIR [8]. Small ncRNA, particularly miRNAs, have extensively been studied for the function in carcinogenesis and the diagnostic and therapeutic potentials in a large variety of malignances [6]. For example, the determination of differential expressions of miRNAs has potential values for diagnosing lung cancer and predicting overall survival of the patients [9]. We previously demonstrated that miRNA expressions could be reliably determined in sputum [10-16]. We recently identified a panel of three sputum miRNA biomarkers (miR-21, 31, and 210) that might be useful in diagnosis of NSCLC [10-16].

New and unexpected functions of middle-size ncRNAs, particularly snoRNAs, have recently been discovered, which may have highly and actively diverse roles in the processes of carcinogenesis than previously thought [17-21] [17, 20, 22, 23] [19, 24]. For instance, we have shown that snoRA42 has oncogenic function in the development and progression of NSCLC [17, 22]. Upregulation of snoRA42 could contribute to lung tumorigenesis by regulating features of tumor-initiating cells [17]. We recently used a GeneChipR Array to analyze snoRNA changes in stage I NSCLC tissues and the matched normal lung tissues [23]. Six snoRNAs (snoRD33, snoRD66, snoRA73B, snoRD76, snoRD78, and snoRA42) were identified whose changes were associated with lung cancer [23]. Based on the previous findings, here we aimed to evaluate if the lung cancer-associated snoRNAs could be used as potential biomarkers for NSCLC.

## RESULTS

### The characteristics of subjects and sputum samples

We recruited lung cancer patients and cancer-free smokers in the University of Maryland Medical Center and the Baltimore VA Medical Center. Of the 256 individuals recruited, 126 were diagnosed with stage I or II NSCLC, and 130 were cancer-free smokers. Following the paradigm for biomarker development that was established by the NCI-Early Detection Research Network [25], we randomly split the 256 cases into a training set and an internal testing set. The training set comprised of 59 NSCLC patients and 61 cancer-free smokers (Table 1). The 59 NSCLC patients had a median age of 66.9 years. Thirty-nine (66.1%) were men and 32 (54.2%) were White Americans. Twenty-nine (51.8%) NSCLC patients were diagnosed with stage I NSCLC, while 30 (48.2%) with stage II NSCLC. Thirty-one (52.5%) NSCLC patients were diagnosed with AC and 28 (47.5%) with SCC. The NSCLC patients were smokers with a median of 45.3 pack-years of smoking. The 61 cancer-free smokers had a median age of 65.7 years and a median of 43.4 pack-years of smoking. Forty (65.6%) were men and 33 (54.1%) were White Americans. The cancer-free smokers were diagnosed with granulomatous inflammation (*n* = 30), nonspecific inflammatory changes (*n* = 21), or lung infections (*n* = 10). The testing set consisted of 67 NSCLC patients and 69 cancer-free smokers (Table 2). The 67 NSCLC patients had a median age of 65.9 years. Forty-five (67.2%) were men and 48 (71.6%) were White Americans. Thirty-three (49.2%) were diagnosed with stage I NSCLC, while 34 (50.8%) with stage II NSCLC. Thirty-five (52.2%) NSCLC patients were diagnosed with adenocarcinoma (AC) and 32 (47.8%) with squamous cell carcinoma (SCC). All the NSCLC patients were smokers with a median of 44.7 pack-years of smoking. The 69 cancer-free smokers had a median age of 64.6 years and a median of 44.4 pack-years of smoking. Forty-six (66.7%) were men and 49 (71.0%) were White Americans. The cancer-free smokers were diagnosed with granulomatous inflammation (*n* = 35), nonspecific inflammatory changes (*n* = 19), or lung infections (*n* = 15). There was no statistically significant difference of the age, race, PNs, forced expiratory volume in 1 second (FEV1), and smoking status between the cases and controls in the two cohort study (All *p* > 0.05) (Tables 1-2).

**Table 1:** Characteristics of lung cancer patients and cancer-free smokers of a training set

	NSCLC cases (n = 59)	Controls (n = 61)	P-value
Age	66.86 (SD 8.93)	65.73 (SD 10.64)	0.28
Sex			0.39
Female	20	21	
Male	39	40	
Race			0.26
White	32	33	
African American	17	18	
Pack-years	45.28 (Range, 31-79)	43.38 (Range, 32-78)	0.69
Nodule size (cm)	3.28 (Range, 0.4-6.3)	3.56 (Range, 0.4-72)	0.54
FEV1/FVC	0.57 (SD, 5)	0.46 (SD, 8)	0.25
Stage			
I	29		
II	30		
Histological type			
AC	31		
SCC	28		

Abbreviations: FEV1, forced expiratory volume in 1 second that represents pulmonary functions;

AC, adenocarcinoma; SCC, squamous cell carcinoma.

**Table 2:** Characteristics of lung cancer patients and cancer-free smokers of a testing set

	NSCLC cases (n = 67)	Controls (n = 69)	P-value
Age	65.90 (SD 8.73)	64.62 (SD 11.59)	0.25
Sex			0.33
Female	22	23	
Male	45	46	
Race			0.12
White	48	49	
African American	19	20	
Pack-years	44.67 (Range, 30-82)	44.36 (Range, 33-78)	0.65
Nodule size (cm)	3.46 (Range, 0.5-6.4)	3.29 (Range, 0.4-6.6)	0.48
FEV1/FVC	0.52 (SD, 6)	0.49 (SD, 7)	0.37
Stage			
I	33		
II	34		
Histological type			
AC	35		
SCC	32		

Abbreviations: FEV1, forced expiratory volume in 1 second that represents pulmonary functions;

AC, adenocarcinoma; SCC, squamous cell carcinoma.

Of the 256 participants (126 NSCLC patients and 130 cancer-free smokers), 48 (18.8%) couldn't spontaneously expectorate sputum, and thus underwent sputum induction by using a Lung Flute. Using the Lung Flute, all the 48 individuals were able to produce sputum. The median volume of sputum collected was 2.7 ml, and the median cell number per ml was 2.7 X10^7^ in each sputum sample. All sputum samples were expelled from the lower respiratory tract, since they had less than 4% oral squamous cells and more than 50% alveolar macrophages. Furthermore, there was no statistical difference of sputum volume, cell number per ml, and percentages of cell populations between lung cancer cases *vs*. cancer-free controls (All *P* < 0.05). In addition, sputum collected by the Lung Flute displayed comparable features as spontaneously expectorated sputum regarding sputum volume, cell number per ml, and percentages of cell populations. Therefore, the specimens were suitable for the downstream cytology and molecular analysis in this study.

### Evaluating analytical performance of quantitative reverse transcription polymerase chain reaction (qRT-PCR) for determination of snoRNA expression in sputum

**Figure 1:**
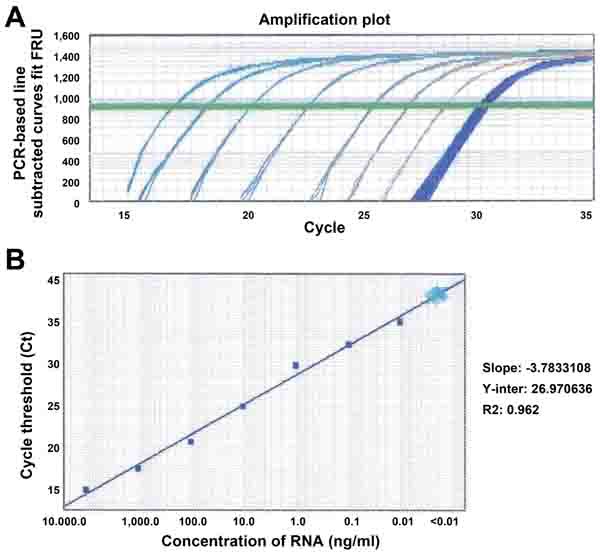
Sensitivity and dynamic range of analysis of snoRD66 in sputum by using qRT-PCR **A.** RNA of sputum was serially diluted in a range of 0.0001 and 10,000 ng/ml. Amplification plot showed that at least seven orders of magnitude (0.01 to 10,000/ml) of sputum RNA were reliably measured. However, the RNA samples with less than 0.01 ng/ml concentration were not able detectable by qRT-PCR demonstrated by more than 35 Ct values and unseparated curves. **B.** Correlation of total RNA input to the threshold cycle values for snoRD66 detected by qRT-PCR assay (R2 = 0.962, slope = −3.78). All experiments were done in triplicates for the analysis of all the six snoRNAs, displaying the same results. The figure only shows the result of the analysis of snoRD66.

**Figure 2:**
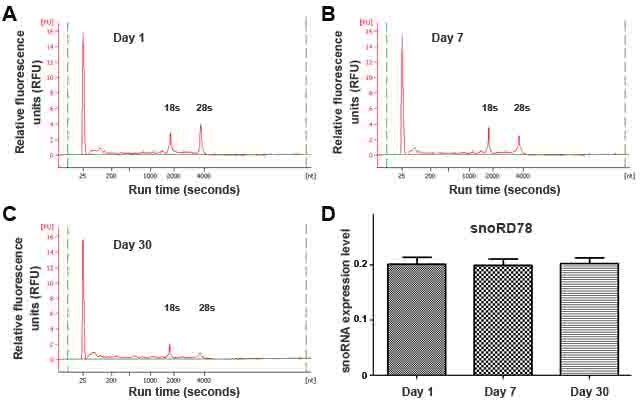
Endogenous snoRNAs were readily and robustly measurable in sputum **A.**-**C.** Analysis of RNA samples by using capillary electrophoresis showed greater degradation in sputum on days 7 and 30 compared with day 1. **D.** The expression levels of snoRD78 measured by using qRT-PCR in the specimens did not change after 30-day storage. miR-16 was used an internal control for normalization of the target snoRNAs. All experiments were done in triplicates for the analysis of all the six snoRNAs, displaying the same results. The figure only shows the result of the analysis of snoRD78.

We previously used a GeneChipR micorarray to analyze snoRNA changes in 22 stage I NSCLC tissues and the matched normal lung tissues [23]. Of the 352 snoRNAs analyzed, 30 were overexpressed and one was underexpressed with ≥ 1.0 fold-change in lung NSCLC tissues compared with the corresponding noncancerous lung tissues (all *P* < 0.05) [23]. Using a predefined criterion of a change ≥2.5-fold, we further identified six snoRNAs that were statistically differently expressed between the paired tumor and noncancerous samples (all *P* < 0.001). The six snoRNAs were snoRD33, snoRD66, snoRA73B, snoRD76, snoRD78, and snoRA42. In this present study, we focused on the six snoRNAs by first determining if they could be reliably detected in sputum. qRT-PCR was employed to determine expression levels of the six snoRNAs in RNA isolated from sputum of ten cancer-free individuals, respectively. All tested snoRNAs had <35 Ct values, indicating that the snoRNAs could easily and readily be measured in sputum. To determine the sensitivity of qRT-PCR assay for quantification of the snoRNAs in sputum, the RNA samples were first diluted at different concentrations (ranged from 0.0001 to 10,000 ng/ml). The serially diluted samples were then tested by using qRT-PCR for determining expression levels of the snoRNAs. The results showed that there was an excellent linearity between the RNA inputs and the Ct values for the snoRNAs tested (Figure 1A). Furthermore, the assay had a dynamic range of more than seven orders of magnitude (R^2^ = 0.962), and was capable of detecting each snoRNAs in as little as 0.01 ng of RNA isolated from sputum (Figure 1B). In addition, the expression levels of the snoRNAs were determined by two research staff. Comparison of the results demonstrated a high degree of correlation (R^2^ = 0.978), suggesting that qRT-PCR assay yielded excellent reproducibility in quantification of snoRNAs in sputum. Moreover, to investigate the stability of snoRNAs in archived sputum, aliquots of ten sputum specimens were stored at 4°C for 1, 7, 30 days, respectively. Bioanalyzer showed that there was increasing degradation of total RNA illustrated by gradually decreased heights of 18S and 28S peaks from day 1 to day 30 (Figure 2A-2C). However, there was no effect on expression levels of snoRNAs as measured by qRT-PCR in the same specimens (Figure 2D). Altogether, expression of the snoRNAs was readily and robustly determined in sputum by using qRT-PCR.

### Developing a panel of sputum snoRNA biomarkers for lung cancer in a training cohort of specimens

**Figure 3:**
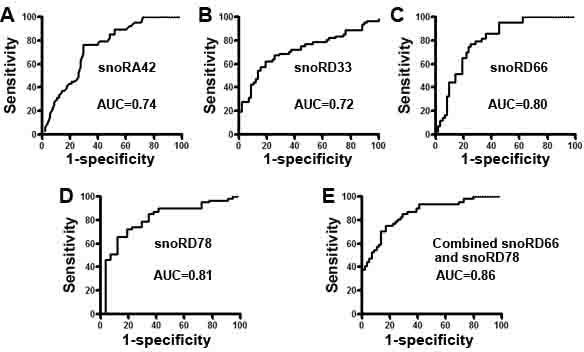
Receiver-operator characteristic (ROC) curve analysis of expression levels of the four snoRNAs in sputum of 59 patients diagnosed with NSCLC and 61 cancer-free smokers The area under the ROC curve (AUC) for each snoRNA conveyed its accuracy in differentiating NSCLC patients from the cancer-free subjects in terms of sensitivity and specificity. **A.**-**D.** The four individual genes resulted in 0.723-0.811 AUC values. **E.** From the four snoRNAs, a small panel of two snoRNAs (snoRD66 and snoRD78) was developed, producing 0.86 AUC, which was significantly higher than that of any single one used alone (All *P* < 0.05).

Four of the six snoRNAs displayed a significantly different level in sputum of NSCLC patients *vs*. cancer-free smokers (all *P* < 0.05) (Table 3) (Supplementary Figure 1). The four snoRNAs were snoRD33, snoRD66, snoRD78, and snoRA42. We used area under the receiver-operator characteristic (ROC) curve (AUC) to determine diagnostic value of each snoRNA. As shown in Table 3, the four snoRNAs exhibited AUC values of 0.723-0.811 in distinguishing lung cancer patients and cancer-free controls. We further used a stepwise logistic regression model to develop an optimal panel of snoRNAs. Two snoRNAs (snoRD66 and snoRD78) were identified as the best biomarkers (all *P* < 0.001). A logisitic regression model with the two snoRNAs was developed as U = −3.632+2.839*log (snoRD66)-1.936*log (snoRD78), where U was the odds of being classified as a case. We calculated the distance to the perfect point (0, 1) with varying cut-offs for U, and the cut-off corresponding to the shortest distance in the AUC analysis was considered the optimal cut-off. The optimal cut-off for the combined biomarkers was U = 0.346. Any subject with U≥0.346 was classified as a NSCLC case. Furthermore, Pearson correlation analysis indicated that the estimated correlations among expression levels of the two snoRNAs in sputum was low (*P* > 0.05), implying that the diagnostic vales of the snoRNAs were complementary to each other. In addition, combined use of the two snoRNAs generated 0.86 AUC, which was significantly higher than that of any single one of the four snoRNAs (Figure 3) (*P* < 0.05). In addition, the analysis of all four snoRNAs in combination did not display a higher AUC value compared with the optimized panel of the two snoRNAs (*P* > 0.05). The panel of two biomarkers didn't exhibit special association with stage and histological type of NSCLC, age, gender, ethnicity, and FEV1 of the participants (All *P* > 0.05). However, the expression level of the snoRNAs was associated with smoking history and size of PN of participants (All *P* < 0.05).

**Table 3:** Expression levels of the snoRNAs in sputum samples of 59 NSCLC patients vs. 61 cancer-free smokers by using qRT-PCR assay

MiRNA	Mean ± SEM in cancer-free controls	Mean ± SEM in NSCLC patients	P value	AUC
snoRA73B	0.3346 ± 0.0335	0.3522 ± 0.0372	0.7276	0.5044 (Std. Error, 0.0366; 95% confidence interval, 0.4328 to 0.5761)
snoRD76	2.173 ± 0.2637	2.5610 ± 0.3279	0.3574	0.5068 (Std. Error, 0.0365; 95% confidence interval, 0.4351 to 0.5784)
**snoRD33**	0.03580 ± 0.0131	0.3330 ± 0.0781	**0.0003**	**0.7230** (Std. Error, 0.0473; 95% confidence interval, 0.6301 to 0.8159)
**snoRA42**	0.1367 ± 0.0179	0.6446 ± 0.1042	**< 0.0001**	**0.7431** (Std. Error, 0.0448; 95% confidence interval, 0.6551 to 0.8310)
**snoRD66**	0.04029 ± 0.0055	0.7112 ± 0.2081	**0.0010**	**0.8065** (Std. Error, 0.0415; 95% confidence interval, 0.7250 to 0.8880)
**sn0RD78**	0.2069 ± 0.03459	0.5529 ± 0.0586	**< 0.0001**	**0.8112** (Std. Error, 0.0408; 95% confidence interval, 0.7311 to 0.8913)

Abbreviations: qRT-PCR, quantitative reverse transcriptase PCR; NSCLC, non-small cell lung cancer;

AUC, the area under ROC curve receiver-operator characteristic curve.

Combined use of the two snoRNAs generated 74.58% sensitivity and 83.61% specificity. Sputum cytology has 45.76% sensitivity and 90.16% specificity. Therefore, the biomarker panel had a significantly higher sensitivity (74.58%) compared with sputum cytology (45.76%), while a low specificity (83.61% *vs*. 90.16%) (All *P* < 0.05). Interestingly, combining both the snoRNA biomarkers and sputum cytology provided a higher sensitivity (81.36%) than any single approach used alone (*P* < 0.05), while still keeping 90.16% specificity.

Since a larger lung PN could more likely to be cancerous compared with a smaller lung PN [26], we used a logistic model analysis to evaluate the integration of size of PNs and biomarker panel for lung cancer diagnosis. The integration produced 0.93 AUC (Supplementary Table 1), which was significantly higher than that (0.86 AUC) of the biomarker panel used alone (*P* = 0.02). Accordingly, combined analysis of the biomarker panel and size of PNs produced both higher sensitivity (85.25% *vs*. 74.58%) and specificity (89.83% *vs*. 83.61%) than did the biomarker panel for lung cancer diagnosis (All *p* < 0.05). Furthermore, because smoking is a main cause of lung cancer, we also evaluated the value of integrating smoking status and the biomarker panel for lung cancer diagnosis. The incorporation of smoking pack-years into this snoRNA biomarker-based model created an AUC of 0.92, being significantly higher than that of the biomarkers (0.86 AUC) (*P* = 0.03) (Supplementary Table 1). Consequently, combined use of the biomarker panel and smoking status produced a higher sensitivity (83.61% *vs*. 74.58%; *P* < 0.05) compared with the biomarker panel used alone, while maintaining a similar specificity (84.75% *vs*. 83.61%; *P* > 0.05). However, the inclusion of both smoking pack-years and size of PNs into this biomarkers model did not exhibit a significantly higher AUC (0.93) than did either integrating the biomarkers with smoking status (0.92) or integrating the biomarkers with size of PNs (0.93) (All *P* > 0.05) (Supplementary Table 1).

In this project, the cases and controls were recruited in the University of Maryland Medical Center and the Baltimore VA Medical Center. Regular CT imaging was performed for all the cases and controls as part of clinical standard care. The initially regular CT scan could identify 55 of the 59 lung cancer cases in the training set, and classified 14 of the 61 cancer-free subjects as lung cancer patients. The CT diagnosis has a sensitivity of 93.22% and a specificity of 77.05% for lung cancer. Therefore, the snoRNA biomarkers had a higher specificity (83.61% *vs*. 77.05%), whereas a lower sensitivity (75.58% *vs*. 93.22%) than did the CT imaging (All *P* < 0.05). Interestingly, combined use of the biomarker panel and CT produced a significantly higher specificity (86.89% *vs*, 77.05%; *P* < 0.05) and a similar sensitivity (91.53% *vs*. 93.22%; *P* > 0.05), as compared with the initial CT scan (Supplementary Figure 2). The observations suggest that the sputum snoRNA biomarkers might have the potential to improve CT scan for lung cancer diagnosis by increasing its specificity.

### Validating the panel of sputum snoRNA biomarkers in a testing cohort of specimens

The panel of sputum snoRNA biomarkers was validated in a testing cohort (Table 2) in a blinded fashion using the optimal thresholds established in the above training set. The panel of the snoRNAs had 74.63% sensitivity and 84.06% specificity for diagnosis of NSCLC. Furthermore, sputum cytology showed 44.78% sensitivity and 91.30% specificity. The use of the sputum biomarkers and cytology in combination produced a higher sensitivity (82.09%) than an approach used alone (All *P* < 0.05), while maintaining 91.30% specificity. In addition, combined analysis of the biomarker panel and size of PNs produced a higher sensitivity (85.07% *vs*. 74.63%) and a higher specificity (89.86% *vs*. 84.06%) compared with the biomarker panel (All *P* < 0.05). Moreover, the integration of the biomarkers and smoking status created a higher sensitivity (83.58% *vs*. 74.63%; *P* < 0.05) compared with the biomarker panel, while keeping a similar specificity (84.06% *vs*. 83.61%; *P* > 0.05). The initially regular CT scan had a sensitivity of 92.54% and a specificity of 76.81% for lung cancer diagnosis. Combined use of the biomarker panel and CT yielded a higher specificity (86.96% *vs*. 76.81%; *P* < 0.05) and a similar sensitivity compared with the initial CT scan (91.04% *vs*. 92.54%; *P* > 0.05). Therefore, the results created from the validation study in a different set of cases and controls confirmed the potential of using snoRNAs as sputum biomarkers for NSCLC.

## DISCUSSION

To the best of our knowledge, this might be the first study to demonstrate that snoRNAs, middle-size ncRNAs, remain intact and are readily detectable in sputum. Furthermore, we developed a small panel of snoRNA biomarkers that had a higher sensitivity compared with sputum cytology for lung cancer diagnosis. In addition, integrating smoking history or size of PNs into the biomarkers further elevated the diagnostic value for lung cancer. Therefore, the analysis of snoRNAs may provide an approach to potentially improve diagnosis of lung cancer.

Accumulated evidences have supported that dysfunction of snoRNAs plays an important role in carcinogenesis [19, 21, 27-29]. For example, we previously found that snoRA42 downregulation restrained NSCLC cell growth *in vivo* and *in vitro* [22]. snoRA42 had oncogenic function in the development and progression of NSCLC by reducing apoptosis of NSCLC cells in a p53-dependent manner [22]. We further showed that upregulation of snoRA42 contributed to lung tumorigenesis by regulating features of tumor-initiating cells [17]. Donsante et al. demonstrated that adeno-related viruses could integrate their genome into mouse genome, causing liver cancer in the animals [30]. Interestingly, the integration sites identified in cancer cells were all located within a DNA interval encoding snoRNAs [30]. Furthermore, upregulation of gas5-generated snoRNAs contributed to an arrest of cell growth [31], and was associated with growth arrest of breast cancer cells [32, 33]. In addition, snoRD 115 regulated splicing of serotonin receptor 2C [34], and hence contributing to posttranscriptional gene silencing. Moreover, as miRNAs, some snoRNAs are located at a chromosomal breakpoint involved in human carcinogenesis. For instance, snoRD50 was originally discovered from the breakpoint of chromosomal translocation t (3,6) (q27;q15), which was involved in human B-cell lymphoma [35]. Small RNA sequences derived from snoRNAs were proposed to function like miRNAs [36]. It is well known that the genes situated at chromosomal genomic amplification regions might have oncogenic function involved in the promotion of cancer [37]. Interestingly, the snoRNAs that are identified to have the potential as biomarkers for NSCLC are located in commonly frequent genomic amplified regions in lung cancer [38, 39]. snoRD66 is located in chromosome 3q27.1 [39], while snoRD78 in chromosomal regions 1q25.1. Both 1q25.1 and 3q27.1 are the most frequently amplified chromosomal segments that may contain potential oncogenes in NSCLC [39]. Our ongoing study is to investigate the biological relevance of the snoRNAs in tumorigenesis.

Lung cancer-related molecular changes might also associate with chronic inflammatory lung diseases [40]. The development of such molecular alterations as biomarkers may produce false positive diagnostic rate for lung cancer. To identify sputum snoRNAs whose changes are specific to lung cancer, here we design a cases-control study, in which, lung cancer patients and cancer-free controls are matched 1:1 by age, gender, race, FEV1, et al. This nested case-control study allows removing confounding effects of the factors on the changes of snoRNAs, and hence identifies the biomarkers that can specifically differentiate lung cancer patients from the controls. Therefore, the snoRNAs might be promising biomarkers for NSCLC, since the expression levels in sputum are independent of these elements. Moreover, no significant difference of the snoRNA expression level was observed between different stages (I and II) of NSCLC, implying that the snoRNAs might be used as potential biomarkers for lung tumors at relatively early stages. In addition, the elevated sputum expression levels of the snoRNAs had equal frequency between AC and SCC of the lungs, suggesting the potential of the snoRNAs as biomarkers for the two major histological types of lung cancer.

The study has some weakness. First, the sensitivity (74.58%) and specificity (83.61%) of the snoRNAs are not sufficient for routine clinical application. To overcome the difficulty, we need to identify additional cancer-associated ncRNAs that can be added to the current ones so that the diagnostic efficacy of the sputum-based assay could be improved. To that end, we are using next-generation deep sequencing to analyze lung tumor specimens for identifying additional NSCLC-related ncRNAs that may provide new biomarker candidates. Furthermore, we could also integrate the snoRNA biomarkers with the other types of biomarkers to improve diagnostic accuracy of the noninvasive approach. For instance, we recently developed a panel of three sputum miRNA biomarkers (miRs-21, 31, and 210) that produced 82.93% sensitivity and 87.84% specificity for lung cancer [10]. Our ongoing efforts are to directly compare the panel of snoRNAs and the set of miRNAs to determine which have a better diagnostic value, and if they have a synergetic value in diagnosis of NSCLC. Second, sputum specimens were collected from the hospital-based patients with clinical diagnosis. The participants might not representative of the heavy smokers in screening setting for lung cancer. We will perform a prospective and multisite lung cancer screening trial to validate the diagnostic value of the biomarkers. Third, the NLST indicated that the early diagnosis of lung cancer by using LDCT could considerably reduce the mortality [2]. Yet LDCT has a low specificity for the early detection of lung cancer, presenting a major clinical challenge [3]. Although our present study shows that integrating the snoRNA biomarkers with CT could produce a higher specificity compared with the CT used alone, the elevated specificity is not sufficient to be used in the laboratory settings. We are designing a new project to develop a more accurate biomarker panel that may dramatically improve the specificity of CT for lung cancer diagnosis by helping distinguish malignant from benign PNs.

In sum, we demonstrated that the snoRNAs existed in a stable form and were readily measurable in sputum. The analysis of sputum snoRNAs may provide a potential tool for diagnosis of NSCLC. The sputum snoRNA biomarkers may be developed as a screening tool for lung cancer in high-risk patients. For example, future use of the biomarkers may complement CT screening for lung cancer by improving diagnosis of NSCLC among CT-discovered indeterminate PNs. Nonetheless, a large multi-center clinical project to further validate the full utility is required before it could be adopted in routine clinical setting.

## MATERIALS AND METHODS

### Patient cohorts

The study protocol was approved by the Institutional Review Boards of the University of Maryland Medical Center and the Baltimore VA Medical Center. All lung cancer patients and control subjects were selected and consented when they visited the clinics of the Division of Pulmonary and Critical Care in the two medical centers. Final diagnoses for the lung cancer patients were confirmed with histopathologic examinations of specimens obtained by CT-guided transthoracic needle biopsy, transbronchial biopsy, videotape-assisted thoracoscopic surgery, or surgical resection. Regular CT imaging was performed as part of clinical standard care using a protocol with a 120-KV, 220-mA tomoscan (model Somatom Plus 4; Seimens; Munich, Germany). The slice thickness was 5 mm through the mediastinum and 8 mm elsewhere. The CT images were read independently by two board-certified radiologists who were blinded to molecular analysis. The two radiologists’ findings were recorded and then discussed, and the consensus findings were documented for study purposes. The diameter of a nodule, as a measure of its size, was defined as the average of its length and width measured with electronic calipers on the image that showed the largest cross-sectional area of the nodule. A positive result of initial CT was defied as previously described [41, 42]. The surgical pathologic staging was determined according to the TNM classification of the International Union Against Cancer with the American Joint Committee on Cancer and the International Staging System for Lung Cancer. Histopathologic classification was determined according to the World Health Organization classification. Control individuals were subjects aged 55-74 had at least a 30 pack-year history of smoking, and had no prior history of any cancer. Furthermore, all control individuals remained cancer free for a minimum 2-year follow-up. The demographic and clinical characteristics of the cases and controls, including stage and histological diagnosis, smoking history, size of PN, and pulmonary functions represented by FEV1 were also collected (Tables 1-2).

### Sputum collection, preparation, and sputum cytology

The subjects were instructed to spontaneously cough sputum as previously described [11-16, 43-52], before receiving any treatment (e.g., surgery, preoperative adjuvant chemotherapy, and radiotherapy). The participants who were not able to spontaneously cough sputum underwent sputum induction using a Lung Flute (Medical Acoustics, Buffalo, NY)-based technique as described in our previous work [13]. Sputum was collected in a sterile cup, and then centrifuged at 1,000xg for 15 min. Cytospin slides were prepared and underwent Papanicolaou staining for evaluating whether the specimens were representative of deep bronchial cells. Cytologic study was performed on the cytospin slides prepared from the sputum samples using the classification of Saccomanno [4]. Positive cytology included both carcinoma *in situ* and invasive carcinoma [10]. Cell pellets from each sample were resuspended in Sputolysin (Calbiochem, San Diego, CA) for 15 minutes at 37°C and then stored at −80°C until being tested.

### Determining expressions of the snoRNAs in sputum by qRT-PCR

RNA was extracted from cell pellets of sputum as previously described [11-16]. The purity and concentration of RNA were determined by OD260/280 readings using a dual beam UV spectrophotometer (Eppendorf AG, Hamburg, Germany). RNA integrity was determined by capillary electrophoresis using the RNA 6000 Nano Lab-on-a-Chip kit and the Bioanalyzer 2100 (Agilent Technologies, Santa Clara, CA). Expressions of the six snoRNAs were determined in sputum by using SYBR green RT-qPCR assay [23]. Briefly, 10 ng of RNA was polyadenylated by poly(A) polymerase and reverse transcribed to cDNA using miScript RT kit (Qiagen, Valencia, CA) according to the manufacturer's instructions. qPCR was performed using miScript SYBR Green PCR kit (Qiagen) with the manufacturer provided miScript Universal primer and the snoRNA-specific forward primers in ABI PRISM 7900 Real-time PCR system (Applied Biosystems, Foster City, CA). The primer sequences for the snoRNAs are shown in Supplementary Table 2. Expression levels of the snoRNAs were calculated using comparative cycle threshold (Ct) method as previously described [10, 11, 14-16]. Ct values of the target snoRNAs were normalized in relation to that of miR-16, which was proven as an internal control for ncRNA quantification in sputum [10]. Relative expression of a targeted snoRNA in a given sample was computed using the equation 2−ΔCt, where ΔCt = Ct (targeted snoRNA) – Ct (miR-16). All assays were performed in triplicates. Furthermore, two interplate controls and one no-template control were carried along in each experiment. The no template control for RT was RNease free water instead of RNA sample input, and no template control for PCR was RNease free water instead of RT products input.

To determine sensitivity and a dynamic range of detecting snoRNAs by RT-qPCR in sputum, RNA isolated from sputum samples of ten cancer-free smokers was diluted in diethyl pyrocarbonate (DEPC) water (Sigma-Aldrich, St. Louis, MO) at different concentrations. The serially diluted RNAs served as experimental samples for measuring expression of each snoRNA. To assess the reproducibility of the RT-qPCR for determination of the snoRNAs in sputum, RNA isolated from the ten sputum samples was analyzed by two research staff. The results were directly compared. To evaluate stability of the snoRNAs in sputum, the ten sputum specimens were divided into 3 parts, respectively. The first aliquot from each sputum specimen was processed immediately for isolating RNA on day 1, while others were stored in 4°C and processed on day 7 and 30. Expression of the snoRNAs was measured by using qRT-PCR in these specimens that were processed from the different time points. All experiments were performed at least three times. The generated data were directly compared.

### Statistical analysis

To identify a panel of biomarkers, we used receiver-operator characteristic (ROC) curve and the area under ROC curve (AUC) to determine sample size of the training set. The AUC of H0 (the null hypothesis) was set at 0.5. H1 represented the alternative hypothesis; accordingly, at least 25 subjects were required in each category to show a minimum difference of interest between an AUC of 0.75 versus an AUC of 0.5 with 90% power at the 5% significance level [53]. Therefore, the sample size of 59 NSCLC patients and 61 cancer-free controls in the training set would provide enough statistical power for the identification of the biomarkers. Furthermore, to estimate sample size of the testing set for the validation of the biomarkers, we also used AUC analysis. The AUC of H0 (the null hypothesis) was set at 0.5. H1 represented the alternative hypothesis. To have a high reproducibility with adequate precision, 60 subjects per group in the testing set were required. With this sample size, we would have 90% power to detect an AUC of 0.75 at the 2% significance level. In addition, we used Pearson's correlation analysis to evaluate the association between snoRNA expressions and demographic and clinical characteristics of the lung cancer patients or cancer-free controls. The clinicopathologic results were used as the reference standards to determine the diagnostic value of each snoRNA biomarker. We used ROC curve and AUC analyses to decide sensitivity, specificity, and corresponding cut-off value of each snoRNA. For each gene, we identified the point on ROC curve that was the closest to the perfect point (0, 1) with sensitivity = 1 and specificity = 1. A stepwise logistic regression model was used to select the optimal panel of snoRNAs [54]. To compare the sensitivities and specificities of the panel of snoRNAs and CT scan used alone, the combination of the snoRNAs and CT, differences between AUC values of each approach were compared as described by Hanley and McNeil [55]. All P values shown were two sided, and a *P* value of <0.05 was considered statistically significant. All analyses, including correlation coefficient, Wilcoxon test, logistic regression, ANOVA, and *t* test, were performed using log transformed data.

## SUPPLEMENTARY MATERIAL TABLES AND FIGURES


