# Supplementary material for: Analysis of small nucleolar RNAs in sputum for lung cancer diagnosis
Source: Oncotarget. 2015 May 20;7(5):5131–42. doi: 10.18632/oncotarget.4219 (PMC4868676; doi:10.18632/oncotarget.4219)
Supplement: Supplementary file 1 [file oncotarget-07-5131-s001.pdf]

Analysis of small nucleolar RNAs in sputum for lung cancer diagnosis

Supplementary Material

Supplementary table 1. Performance of the integration of smoking pack-years or size of PNs with a panel of the two snoRNA biomarkers (snoRD66 and snoRD78) for lung cancer diagnosis

	AUC	Sensitivity	Specificity
The biomarker panel	0.86 (Std. Error, 0.03; 95% confidence interval, 0.79 to 0.92)	74.58%	83.61%
The biomarker panel and size of PNs	0.93 (Std. Error, 0.02; 95% confidence interval, 0.89 to 0.97)	85.25%	89.83%
The biomarker panel and smoking pack-years	0.92 (Std. Error, 0.02; 95% confidence interval, 0.87 to 0.97)	83.61%	84.75%
The biomarker panel, size of PNs, and pack-years	0.93 (Std. Error, 0.02; 95% confidence interval, 0.89 to 0.98)	86.89%	89.23%

PN, pulmonary nodules; AUC, the area under ROC curve receiver-operator characteristic curve.

Supplementary Table 2. The primer sequences for the six snoRNAs

	Forward primer	Reverse primer
snoRD33	5'- ACT TCT CCC ACT CAC ATT C -3'	5'- TGG CCT CAG ATG GTA GTG CAT GTG G -3'
snoRA42	5'- TGG TAA TGG ATT TAT GGT GGG T -3'	5'- GGA CTG GGC AAT GGT TCG -3'
snoRD66	5'- TCT GAT GAC TTC CTG TTA GTG CCA -3'	5'- TTC CTC AGA TCC TCA GTT CCA TCA T -3'
snoRA73B	5'- CCC CAG GCT CTG TCC AA -3'	5'- CGA GGC CCA GCT TCA TT -3'
snoRD76	5'- TGCCACAATGATGACAGTTTATTTG -3'	5'- GCCTCAGTTAAGATAATGGTGGTT -3'
snoRD78	5'- GTG TAA TGA TGT TGA TCA AAT GTC T -3'	5'- TAC CTT TGT CTA CAT GCT CAT TTC A -3'

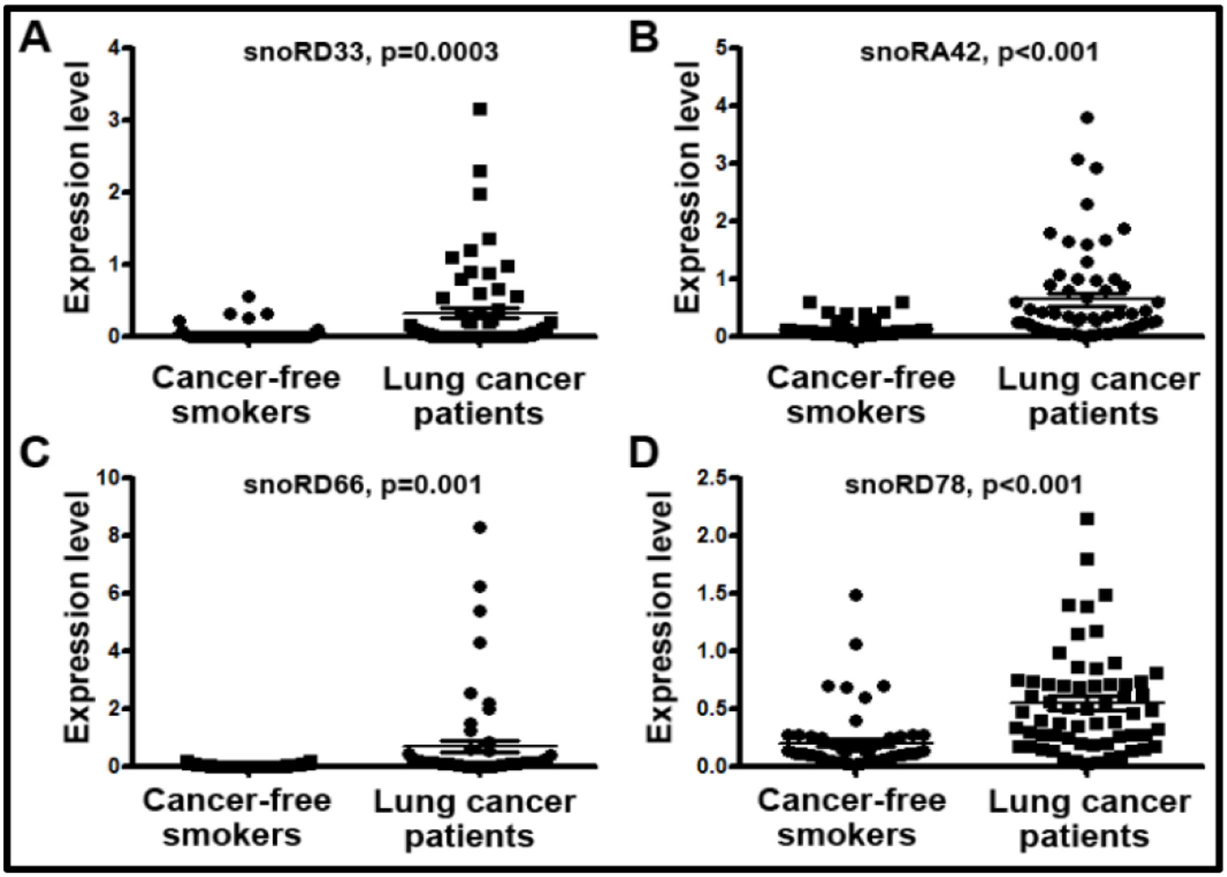

Supplementary Fig. 1. Comparison of the four snoRNAs that show statistical expression difference in sputum in 59 cancer-free smokers *vs*. 61 lung cancer patients. Horizontal lines indicate mean values.

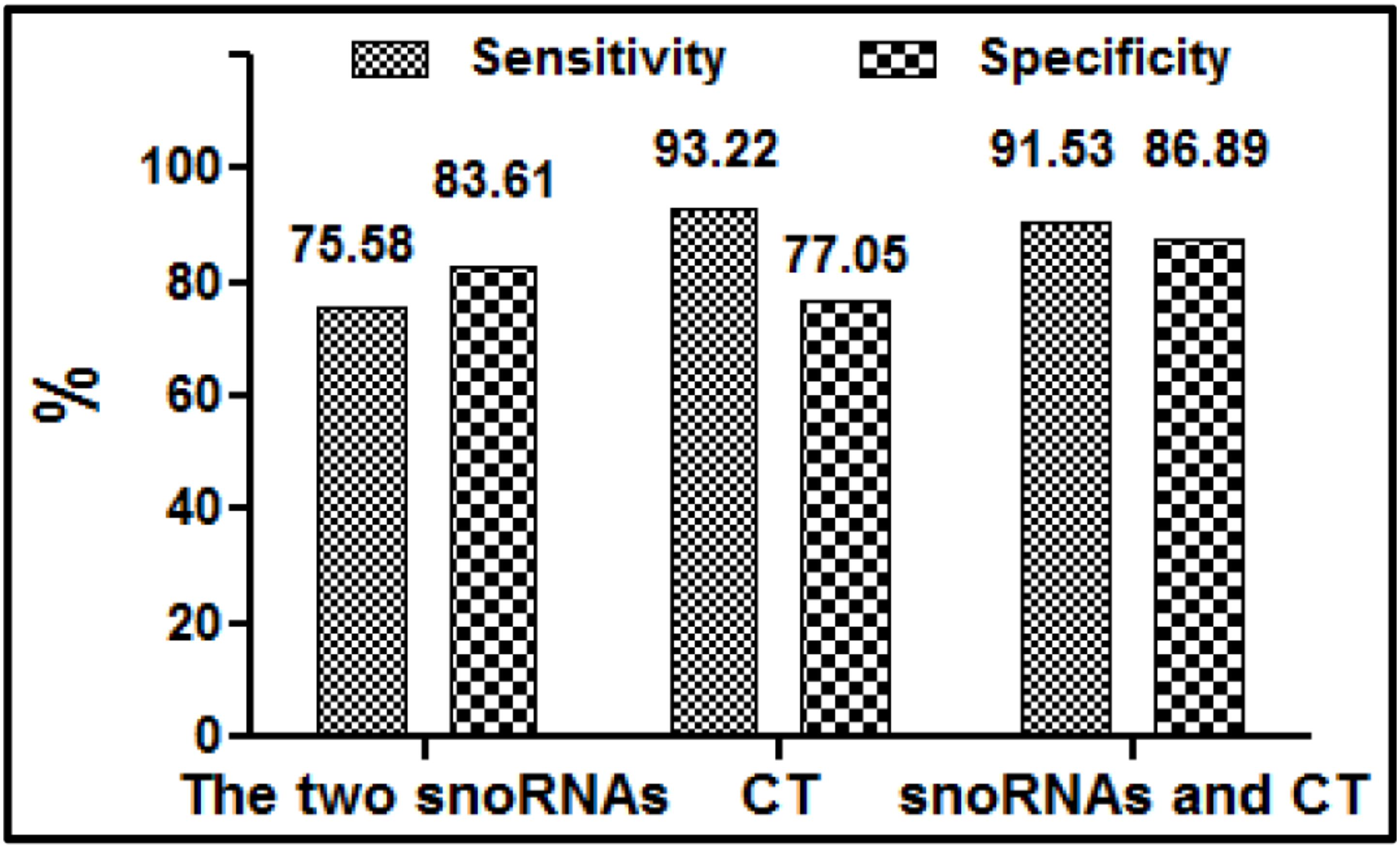

Supplementary Fig. 2. The biomarker panel improves specificity of CT for lung cancer diagnosis. Combined use of the snoRNAs produces 75.58% sensitivity and 83.61% specificity. CT produces 93.22% sensitivity and 77.05% specificity. CT has a lower specificity and a higher sensitivity compared to the panel of the sputum biomarkers (All $p<0.05$). Integrating the miRNAs and CT yields a higher specificity (86.89% *vs*. 77.05%; $P<0.05$) and a similar sensitivity compared with the initial CT scan used alone (91.53% *vs*. 93.22%; $P>0.05$).
